# Association between Sleep Duration, Insomnia Symptoms and Bone Mineral Density in Older Boston Puerto Rican Adults

**DOI:** 10.1371/journal.pone.0132342

**Published:** 2015-07-06

**Authors:** Jinya Niu, Shivani Sahni, Susu Liao, Katherine L. Tucker, Bess Dawson-Hughes, Xiang Gao

**Affiliations:** 1 Department of Epidemiology and Bio-statistics, Institute of Basic Medical Sciences, Chinese Academy of Medical Sciences/ Peking Union Medical College, Beijing, China; 2 Institute for Aging Research, Hebrew SeniorLife, Beth Israel Deaconess Medical Center, Harvard Medical School, Boston, Massachusetts, United States of America; 3 Department of Clinical Laboratory and Nutritional Sciences, University of Massachusetts Lowell, Boston, Massachusetts, United States of America; 4 Bone Metabolism Laboratory, Jean Mayer USDA Human Nutrition Research Center on Aging at Tufts University, Boston Massachusetts, United States of America; 5 Department of Nutritional Science, The Pennsylvania State University, University Park, Pennsylvania, United States of America; University of Rome Tor Vergata, ITALY

## Abstract

**Objective:**

To examine the association between sleep patterns (sleep duration and insomnia symptoms) and total and regional bone mineral density (BMD) among older Boston Puerto Rican adults.

**Materials/Methods:**

We conducted a cross-sectional study including 750 Puerto Rican adults, aged 47–79 y living in Massachusetts. BMD at 3 hip sites and the lumbar spine were measured using dual-energy X-ray absorptiometry. Sleep duration (≤5 h, 6 h, 7 h, 8 h, or ≥9 h/d) and insomnia symptoms (difficulty initiating sleep, difficulty maintaining sleep, early-morning awaking, and non-restorative sleep) were assessed by a questionnaire. Multivariable regression was used to examine sex-specific associations between sleep duration, insomnia symptoms and BMD adjusting for standard confounders and covariates.

**Results:**

Men who slept ≥9h/d had significantly lower femoral neck BMD, relative to those reporting 8 h/d sleep, after adjusting for age, education level, smoking, physical activity, depressive symptomatology, comorbidity and serum vitamin D concentration. This association was attenuated and lost significance after further adjustment for urinary cortisol and serum inflammation biomarkers. In contrast, the association between sleep duration and BMD was not significant in women. Further, we did not find any significant associations between insomnia symptoms and BMD in men or women.

**Conclusions:**

Our study does not support the hypothesis that shorter sleep duration and insomnia symptoms are associated with lower BMD levels in older adults. However, our results should be interpreted with caution. Future studies with larger sample size, objective assessment of sleep pattern, and prospective design are needed before a conclusion regarding sleep and BMD can be reached.

## Introduction

Osteoporosis and low bone mass are currently estimated to be a major public health threat for almost 53.6 million US adults aged ≥50 y [1]. Although most of the individuals in the US with osteoporosis or low bone mass are non-Hispanic white women, a substantial number of men and women from other racial/ethnic groups also have osteoporosis [2]. This reduction in bone density markedly increases the risk of skeletal fractures, and the consequent pain and loss of function impinge adversely on quality of life. In the United States and the European Union, approximately 30% of all postmenopausal women have osteoporosis, and it has been predicted that more than 40% of them may suffer one or more fragility fractures during their remaining lifetime [2].

By 2025, annual fractures and associated costs are expected to rise by 50%, from $17 billion in 2005 [3]. The most rapid increase is projected to occur in Hispanic and other sub-populations. The annual cost for fractures in Hispanics is expected to grow from $754 million in 2005 to more than $2 billion per year by 2025—an increase of 175% [3]. In this context, identification of potential risk/protective factors for osteoporosis and related disorders in this population has important public health implications.

In parallel with osteoporosis, insomnia and disturbed sleep disproportionally affect older adults [4]. Some studies have shown that insufficient sleep and insomnia could increase the risk of obesity, diabetes and cardiovascular diseases, by elevating insulin resistance and pro-inflammatory responses [5–7]. In particular, sleep deprivation has been found to be associated with increased cortisol concentration during the night [7, 8]. Excess glucocorticoids may contribute to decreased bone formation and lower bone mineral density (BMD) [9]. Further, some studies have shown that short sleep or prolonged sleep (compared to 8h) is associated with an increase in the production of pro-inflammatory cytokines, such as C-reactive protein (CRP), and interleukin 6 (IL-6) [10–14], which increase osteoclast activity and consequent bone loss [15, 16]. Only few population-based studies have examined the association between sleep duration and BMD [17–21] and none have examined the association between insomnia symptoms and BMD. Furthermore, people with insomnia disorder are more likely to have short sleep duration. Therefore, sleep duration and insomnia disorder may have joint effects on BMD, though no previous study has examined this.Hence, the information on whether sleep duration or insomnia symptoms can influence BMD remains unclear.

Previous research in Hispanics has focused primarily on Mexican Americans, because of their majority as a Hispanic subgroup in the US. However, Puerto Ricans are the largest Hispanic subgroup in the northeastern US, and prior research indicates that they have established health disparities and a greater burden of chronic diseases than Mexican Americans [22]. Yet, there is a paucity of research on bone health in this population.

Therefore, in the current study, we examined cross-sectional associations between sleep patterns (sleep duration and insomnia symptoms) and total and regional BMD in older Boston Puerto Ricans adults. We further examined if these associations are mediated by pro-inflammatory cytokines (CRP, and IL-6) and cortisol concentrations. Lastly, we examined the joint effects of sleep duration and insomnia symptoms on BMD.

## Methods

### 2.1. Participants

We used data from the Boston Puerto Rican Osteoporosis Study, an ancillary study of the Boston Puerto Rican Health Study, which is an ongoing cohort of older Puerto Ricans (aged 45-75y at baseline) living in the greater Boston area. The study was approved by the Institutional Review Board at Tufts Medical Center and Northeastern University. All participants provided written informed consent. The design of the Boston Puerto Rican Health Study has been described elsewhere [23]. Briefly, at the baseline (June 2004 to October 2009) and at 2-year follow-up, bilingual interviewers visited the participants’ homes and administered questionnaires to collect information on socioeconomic status, health and health behaviors, acculturation, depressive symptoms, stress, social support, usual diet, and cognitive function. In addition, blood pressure, anthropometric and physical performance measures were collected. Biological samples, including saliva, urine, and 12-h fasting blood, were collected by the study phlebotomist in the participants’ homes on the day after the interview or as soon as possible thereafter.

At the completion of the 2-y follow-up, participants were invited to participate in the Osteoporosis Study. An appointment was made for consenting participants to visit the Metabolic Research Unit at the Human Nutrition Research Center on Aging at Tufts University to undergo bone density and body-composition measurements, to have additional blood samples collected, and to complete additional questionnaires on osteoporosis medication use. By September 2010, 756 of a total of 1,123 participants who completed 2-y follow-up visits consented to the Osteoporosis Study. Primary reasons for non-participation included not being interested in the Osteoporosis Study (n = 163), scheduling problems (n = 139), loss to follow-up (n = 33), and relocation out of Massachusetts (n = 15). An additional, 17 participants died since their 2-y follow-up interview. We further excluded 6 participants without complete data on BMD or sleep. Our final analytic sample included 750 participants (207 men and 543 women) in the current study.

Individuals who declined were more likely to be older (60.2 y compared with 58.7 y; P = 0.002), to have lower BMI (31.1 Kg/m^**2**^ compared with 32.2 Kg/m^**2**^; P = 0.004) and to have a lower score on the Center for Epidemiologic Studies Depression Scale (CES-D; 16.7 compared with 18.9; P = 0.004). No significant differences were observed in other socio-demographic data.

### 2.2. Outcome assessment

We measured BMD (g/cm^2^) at the femoral neck, trochanter, total hip, and posterior-anterior lumbar spine (L2-L4) by DXA (Lunar model Prodigy scanner; General Electric) using standard procedures. The root mean square precision of these measurements were 0.65% for total-hip BMD, 1.03% for the trochanter, 1.31% for the femoral neck, and 1.04% for the lumbar spine [24]. During the study, the stability of DXA measurements was determined by scanning an external standard (aluminum spine phantom; Lunar Radiation Corp) every week.

### 2.3. Exposure assessment

Sleep duration and insomnia symptoms were collected with an additional questionnaire within 1 month, or as soon as possible thereafter, of the 2-y follow-up visit for the parent study. All questionnaires were administered by trained bilingual interviewers. Sleep duration was determined from the question, “please indicate the total number of hours that you sleep, typically, during a 24 hour period?”, with six possible response: 5h or less, 6h, 7h, 8h, 9h and 10h or more. Because of few participants in the 10+h category (12 men and 32 women), we combined it with the 9h category.

Insomnia symptoms were collected with the following questions: “How frequently do you have difficulty falling asleep? (difficulty initiating sleep)”, “How frequently do you have trouble with waking up at night? (difficulty maintaining sleep)”, “How frequently do you have trouble with waking up too early in the morning and not being able to fall asleep again? (early-morning awaking)”, “How frequently do you feel truly tired when you wake up in the morning? (non-restorative sleep)”. The answers were given in three categories: “most of the time”, “sometimes”, “almost never or never”. For this study, insomnia symptoms were defined as (most of the time versus sometimes or rarely/never) in the analysis. A participant who reported most of the time for having difficulty initiating sleep, difficulty maintaining sleep or early-morning awakening, accompanied with most of the time for non-restorative sleep, was considered to have insomnia disorder [25].

### 2.4. Assessment of covariates

At the 2-y follow-up visit, information on age, sex, educational level, snoring, drinking, smoking status, comorbidity and osteoporosis medication use was collected by questionnaire. Participants were categorized as former drinker, current moderate drinker (≤1 drink per day for women or ≤2 drinks per day for men), or heavy drinker (> moderate daily drinking, or binge drinking >6 drinks during one day of drinking. Smoking status was categorized as never (<100 cigarettes in entire life), former, or current smoker. Information on osteoporosis prescription medication use (including calcitonin, calcium, bisphosphonates, vitamin D, and cod liver oil) was collected. Physical activity was assessed with a modified Paffenbarger questionnaire from the Harvard Alumni Activity Survey [26, 27]. To measure symptoms of depression and anxiety, we administered the CES-D Scale, which has been widely used in epidemiologic studies and has been shown to have good consistency and validity in older adults [28]. The CES-D scores range from 0 to 60, with higher scores indicating high severity of depression symptoms.

Serum and urine measures: Fasting blood samples (12h) were drawn from participants by a certified phlebotomist during the morning of the osteoporosis study visit. Plasma 25-hydroxyvitamin D (ng/mL) was measured from fasting blood samples (12h) using an Iradioimmuno assay kit procedure (DiaSorin Inc), as specified by the manufacturer’s procedural documentation (68100E). The intra- and inter-assay CVs were 10.8% and 9.4%, respectively. High sensitivity CRP was measured by a solid-phase, enzyme-labeled chemiluminescent immunometric assay [Immulite 1000, Diagnostic Products Corporation (DCP) Los Angeles, CA 90045–5597] as specified by the manufacturer’s procedural documentation (PILKCR-7, 2003-11-25). Serum IL-6 concentration was measured with a non-cross-reacting enzyme-linked immunoassay (ELISA), employing specific monoclonal and polyclonal antibodies for the analysis of specific cytokine antigens (Quantikine ELISA, R&D Systems, Minneapolis, MN, USA). A 12-hour overnight urine sample was collected following the in-home interview. Urinary cortisol was analyzed by direct immunoenzymatic colorimetric method with an ALPCO cortisol assay (ALPCO, Windham, NH), which is standardized by multiplying each measure by total 12-hour urine volume and dividing by urinary creatinine excretion.

### 2.5. Statistical analyses

All statistical analyses were performed using SAS version 9.2 (SAS Institute Inc.). Formal hypothesis testing was 2-sided, and the nominal type I error rate was 0.05. Because the distribution of sleep and many covariates are sex-specific, we stratified all analyses by sex.

We used the general linear regression models to estimate beta-coefficients and 95% confidence intervals for the association between each of the following exposures: sleep duration (in categories), each insomnia symptom (yes/no), insomnia disorder (yes/no), and each of the BMD outcomes (BMD at femoral neck, trochanter, total hip, and lumbar spine as continuous variables). Because previous studies have reported that short or prolong sleep duration, compared with normal sleep duration (generally 8 h/d) is associated with unfavorable health outcomes [29, 30], we used 8 h/d as the reference category for sleep duration. We also examined joint effects of insomnia disorder (yes/no) and sleep duration (≤5h/d, 6h/d, 7h/d, 8h/d or ≥ 9h/d) on BMD with 8h/d accompanied with no insomnia disorder as the reference category.

In the multivariable model, we adjusted for age (years), education level, smoking status (never, former or current), drinking status (non-drinker/former drinker, current moderate drinker and current heavy drinker), BMI (Kg/m^2^), physical activity score, obesity (BMI≥30 Kg/m^2^), hypertension (yes/no), diabetes(yes/no), arthritis (yes/no), plasma 25-hydroxyvitamin D concentration (ng/mL), snoring frequency (every/most nights, a few nights a week, and occasionally/rarely), osteoporosis medication use (yes/no) and CES-D score. For women, we also adjusted for menopausal status (yes/no).

To understand the potential role of inflammation/cytokines in the pathway linking sleep and BMD, we further adjusted the models for urinary cortisol, serum CRP and IL-6 concentrations. We conducted a test for interaction of insomnia symptoms and sleep duration separately with age, BMI, urinary cortisol, serum CRP and IL-6 concentrations, in relation to BMD. For the interaction test, multiplicative terms were added to the the full models listed above.

We performed two sensitivity analyses: 1) we excluded participants with frequent snoring (every night or most nights); and 2) we further adjusted the models for dietary intake of calcium (mg/d), vitamin C (mg/d), total energy (Kcal/d), and of fruit and vegetables (servings/day).

## Results

### 3.1. Basic characteristics

The proportion of individuals reporting difficulty initiating sleep, difficulty maintaining sleep, early-morning awakening, and non-restorative sleep (most of the time) were 39.9%, 37.9%, 41.9% and 23.2%, respectively. The prevalence of insomnia disorder was 16.9% (15.0% in men and 17.7% in women). Men with insomnia disorder were more likely to smoke, have shorter sleep duration, and have higher CES-D score (p<0.005), but they were less likely to drink, compared to those without the disorder. Women with insomnia disorder were more likely to be older, to have shorter sleep duration (p<0.005) and to have higher CES-D score compared to those without insomnia disorder (Table 1).

**Table 1:** Descriptive characteristics of Boston Puerto Rican adults, by sex and insomnia disorder (y/n)^a^.

	Men (n = 207)		Women (n = 543)	
	With Insomnia disorder ^b^	Without insomnia disorder	With Insomnia disorder ^b^	Without insomnia disorder
	n = 31	n = 176	n = 96	n = 447
Age (y)	56.7±6.2^c^	58.5±8.1	57.3± 7.5	59.3±7.2*
Education level (%)				
Less than 5th grade	25.8	16.6	30.2	24.6
5th–9th grade	19.4	26.3	26.0	25.3
9th–12th grade	35.5	44.6	30.2	34.0
College or bachelor’s degree	16.1	10.9	12.5	13.6
At least some graduate school	3.23	1.71	1.04	2.46
BMI (kg/m^2^)	29.6±5.3	30.1±5.3	33.1± 5.9	33.1± 7.0
Height (m)	1.68±0.05	1.68±0.07	1.55±0.06	1.55±0.06
Obesity (%)	46.7	48.6	68.4	65.6
Physical activity score	31.5±3.8	33.1±6.0	30.8± 4.0	31.4± 4.1
Plasma 25(OH)D (ng/mL)	15.8±5.9	18.0±6.6	20.1± 8.0	19.3± 7.1
Calcium intake (mg/d)	858±547	972±521	1029±604	978±537
Smoking status (%)				
Never smoke	22.6	32.2*	47.9	53.0
Past smoker	25.8	38.5	28.1	28.2
Current smoker	51.6	29.3	24.0	18.8
Drinking status (%)				
Not current drinker	71.0	47.0*	63.2	64.9
Current moderate drinker	22.6	38.1	32.6	31.5
Current heavy drinker	6.45	14.9	4.21	3.62
Currently using bone med (%)	29.0	21.0	49.0	51.7
Hypertension (%)	53.3	67.6	62.5	67.6
Diabetes (%)	38.7	40.3	45.8	41.5
Arthritis (%)	45.2	40.9	71.9	66.9
Sleep duration (%)				
≤5h	45.2	22.4*	45.8	21.5*
6h	19.3	21.3	25.0	23.4
7h	19.3	21.3	8.3	21.5
8h	6.5	21.8	14.6	20.9
≥9h	9.7	13.2	6.3	12.7
Snoring frequency (%)				
Every/most night	45.2	40.6	37.2	38.7
a few nights a week	16.1	18.3	12.8	14.4
occasionally/rare	38.7	41.1	50.0	46.9
CES-D score	24.9± 12.8	13.6±11.2*	22.8±12.5	19.6±12.5*
Menopause (%)	-	-	84.2	88.0
Urinary cortisol ^d^	41.6±25.8	42.6± 45.4	36.6± 35.8	35.5 ±25.2
CRP (mg/L)	1.87 (1.08,3.23) ^e^	2.96 (2.47,3.53)	3.55 (2.77,4.54)	3.49 (3.13,3.90)
IL-6 (μg/L)	3.61(2.70,4.85)	3.33 (2.82,3.93)	3.25 (2.82,3.75)	3.21 (2.98,3.46)
Femoral neck BMD (g/cm^**2**^)	0.981±0.182	1.002±0.150	0.919±0.123	0.905±0.143
Trochanter BMD (g/cm^**2**^)	0.901±0.179	0.908±0.153	0.791 0.114	0.795±0.140
Total femur BMD (g/cm^**2**^)	1.09±0.18	1.09±0.16	1.01±0.13	1.00±0.16
Lumbar spine BMD (g/cm^**2**^)	1.22±0.21	1.24±0.21	1.12±0.18	1.13±0.18

^**a**^ CES-D, Center for the Epidemiologic Studies Depression Scale; CRP, c-reactive protein; 25(OH)D, 25-hydroxyvitamin D; IL-6, interleukin 6; BMD, bone mineral density.

^**b**^ The category“with insomnia disorder” includes participants who reported having difficulty initiating sleep, difficulty maintaining sleep or early-morning awakenings most of the time, accompanied with non-restorative sleep most of the time.

^**c**^ Mean±SD (all such values).

^**d**^ Urinary cortisol (mg) is standardized by multiplying each measure by total urine volume and dividing by urinary creatinine excretion.

^**e**^ Geometric mean, 95% confidence interval in parentheses (all such values).

* p<0.05 compared to those with insomnia disorder.

### 3.2. Sleep duration, insomnia symptoms and BMD

Men who slept ≥9 h/d had significantly lower femoral neck BMD, relative to those with 8 h/d sleep [beta (95%CI): -0.085(-0.17, -0.0014)], after adjusting for smoking, alcohol drinking, physical activity, plasma vitamin D concentration, snoring frequency, depression, and osteoporosis medication use. Further adjustment for educational level, obesity, hypertension, diabetes and arthritis, did not change results (Table 2). Men who slept ≥9 h/d had significantly lower femoral neck BMD, relative to those with 8 h/d sleep [beta (95%CI): -0.092(-0.18, -0.0086)]. The association was attenuated after further adjustment for urinary cortisol and serum inflammation biomarkers [beta (95%CI): -0.00(-0.17, 0.008)].We did not find any significant association between sleep duration and BMD in women (Table 2).

**Table 2:** Associations between sleep duration and bone mineral density (BMD)^a^.

	Sleep duration ^b^				
	≤5h	6h	7h	8h	≥9h
**Men** (n = 207)	n = 53	n = 43	n = 43	n = 40	n = 26
**Femoral Neck BMD**	-0.00052(-0.069, 0.068)	0.013(-0.055, 0.083)	-0.030(-0.099, 0.038)	_(ref)	-0.092*(-0.18, -0.0086)
**Trochanter BMD**	-0.019(-0.087, 0.049)	-0.040(-0.11, 0.028)	-0.047(-0.11, 0.021)	_(ref)	-0.074(-0.16, 0.0082)
**Total femur BMD**	-0.0085(-0.080, 0.063)	-0.031(-0.10, 0.040)	-0.059(-0.13, 0.012)	_(ref)	-0.086(-0.17, 0.00056)
**Lumbar spine BMD**	-0.00025(-0.091, 0.091)	-0.076(-0.17, 0.015)	-0.090(-0.18, 0.0014)	_(ref)	-0.059(-0.17, 0.052)
**Women** (n = 543)	n = 139	n = 127	n = 103	n = 106	n = 62
**Femoral Neck BMD**	0.00045 (-0.034, 0.035)	-0.025(-0.059, 0.010)	-0.0031(-0.039, 0.034)	_(ref)	-0.013(-0.056, 0.030)
**Trochanter BMD**	-0.0025(-0.035, 0.030)	-0.017(-0.050, 0.017)	-0.014(-0.049, 0.020)	_(ref)	-0.021(-0.062, 0.020)
**Total femur BMD**	-0.0071(-0.043, 0.029)	-0.013(-0.049, 0.023)	-0.015(-0.053, 0.023)	_(ref)	-0.030(-0.075, 0.015)
**Lumbar spine BMD**	-0.024(-0.070, 0.022)	-0.021(-0.068, 0.025)	-0.020(-0.069, 0.029)	_(ref)	-0.023(-0.081, 0.035)

^**a**^ Values in the table are β coefficients and 95% confidence intervals from regression models, with 8h as the reference category.

^**b**^ Adjusted for age (years), educational level, smoking status (never smoke, past smoker and current smoker), drinking status (not current drinker, current moderate drinker and current heavy drinker), body mass index (Kg/m^2^), physical activity score, presence of hypertension (y/n), diabetes (y/n), arthritis (y/n), plasma 25-hydroxyvitamin D concentration (ng/mL), snoring frequency (every/most night, a few nights a week, and occasionally/rare) and osteoporosis medication use (y/n), CES-D score among men, and menopause status(y/n; women alone).

* p<0.05 relative to the reference category (8 h/d).

Neither insomnia disorder nor any of the insomnia symptoms were associated with BMD in either men or women (Table 3). Results did not change after further adjustment for urinary cortisol and serum inflammation biomarkers (P range: 0.08–0.98, data not shown). Interactions of sleep duration or insomnia symptoms with age, BMI, urinary cortisol or inflammatory biomarkers were not significant (P interaction >0.05 for all). We further examined joint effects of insomnia disorder (yes/no) and sleep duration (≤5h/d, 6h/d, 7h/d, 8h/d or ≥ 9h/d) on BMD, but found no significant associations (P range: 0.10–1.00, data not shown).

**Table 3:** Associations between insomnia symptoms and bone mineral density (BMD)^a^.

	Difficulty initiating sleep ^b^		Difficulty maintaining sleep ^b^		Early-morning awaking ^b^		Non-restorative sleep ^b^		Insomnia ^b^	
	No	Yes	No	Yes	No	Yes	No	Yes	no	yes
**Men** (n = 207)	n = 137	n = 70	n = 138	n = 69	n = 129	n = 78	n = 159	n = 48	n = 176	n = 31
**Femoral Neck**	_(ref)	-0.0034(-0.054, 0.047)	_(ref)	0.0016(-0.034 0.066)	_(ref)	0.050(0.0023, 0.097)	_(ref)	0.0074(-0.045, 0.060)	_(ref)	0.0060(-0.062, 0.074)
**Trochanter**	_(ref)	-0.0028(-0.052, 0.046)	_(ref)	0.0022(-0.047, 0.051)	_(ref)	0.035(-0.012, 0.082)	_(ref)	0.0099(-0.042, 0.062)	_(ref)	0.018(-0.049, 0.084)
**Total femur**	_(ref)	-0.0013(-0.053, 0.050)	_(ref)	0.0036(-0.048, 0.055)	_(ref)	0.045(-0.0045, 0.095)	_(ref)	0.018(-0.037, 0.072)	_(ref)	0.028(-0.042, 0.098)
**Lumbar spine**	_(ref)	0.046(-0.020, 0.11)	_(ref)	0.0060(-0.072, 0.060)	_(ref)	0.00062(-0.063, 0.063)	_(ref)	0.0037(-0.066, 0.074)	_(ref)	0.0064(-0.096, 0.083)
**Women** (n = 543)	n = 314	n = 229	n = 327	n = 215	n = 307	n = 236	n = 417	n = 126	n = 447	n = 96
**Femoral Neck**	_(ref)	0.012(-0.012, 0.034)	_(ref)	0.016(-0.0065, 0.040)	_(ref)	-0.0075(-0.031, 0.016)	_(ref)	-0.0028(-0.030,0.024)	_(ref)	0.0047(-0.025, 0.035)
**Trochanter**	_(ref)	0.018(-0.0043, 0.040)	_(ref)	0.0054(-0.017, 0.028)	_(ref)	-0.011(-0.033, 0.011)	_(ref)	-0.011(-0.038, 0.014)	_(ref)	-0.0098(-0.038, 0.019)
**Total femur**	_(ref)	0.0088(-0.015, 0.033)	_(ref)	0.0061(-0.018, 0.030)	_(ref)	-0.015(-0.039, 0.0089)	_(ref)	0.0046(-0.023, 0.033)	_(ref)	-0.0054(-0.037, 0.026)
**Lumbar spine**	_(ref)	0.0092(-0.021, 0.040)	_(ref)	0.0025(-0.028, 0.034)	_(ref)	-0.022(-0.053, 0.0091)	_(ref)	0.0091(-0.027, 0.045)	_(ref)	0.017(-0.023, 0.058)

^**a**^ Values in the table are β coefficients and 95% confidence interval in the regression model.

^**b**^ Adjusted for age (year), educational level, smoking status (never smoke, past smoker and current smoker), drinking status (not current drinker, current moderate drinker and current heavy drinker), body mass index (Kg/m^2^), physical activity score, presence of hypertension (y/n), diabetes (y/n), arthritis (y/n), plasma 25-hydroxyvitamin D concentration (ng/mL), snoring frequency (every/most night, a few nights a week, and occasionally/rare), osteoporosis medication use (y/n), CES-D score among men, and menopause status (y/n, women alone).

### 3.3. Sensitivity analysis

Exclusion of participants with frequent snoring did not change the results (data not shown).

When full models were further adjusted for dietary intakes of calcium, vitamin C, total energy, fruit and vegetables, the results did not change (data not shown).

## Discussion

In this cross-sectional study of 750 older Boston Puerto Rican adults, 16.9% reported insomnia disorder, based on the NIH’s description of insomnia [25]. In a review by Ohayon [31], the prevalence of insomnia related symptoms was 30–48%. However, the prevalence dropped to 12–16% when frequency modifiers were added to symptoms such as presence of symptoms “often” or “always”. This is consistent with our observations.

In the present study, men who slept 9h or more per day had significantly lower femoral neck BMD, relative to those with 8 h/d sleep, after adjusting for age, smoking, physical activity, serum vitamin D, use of osteoporosis medication and other potential confounders. Further adjustment for cortisol and pro-inflammatory cytokine concentrations attenuated these results, suggesting that inflammation may play a role in this pathway. However, given the number of tests conducted in this study and no significant results for other BMD sites, it is unclear whether this significant association was due to chance. We did not find any significant association between sleep duration or insomnia symptoms and BMD among women.

To date, only a few cross-sectional studies have examined the association between sleep duration and BMD among adults, and these have generated mixed results. In one cross-sectional study including 1,146 women and men aged 20–60 years, Specker et al. found that women, but not men, with short sleep duration (<6.5 h/night) had lower cortical volumetric BMD than those with sleep duration >6.5 h/night after adjustment for potential covariates, such as age, weight, height, physical activity, and intakes of fat, calcium and vitamin D [18]. However, they did not find any significant differences in BMD at spine or hip sites between sleep-deprived and sleep-adequate women or men. In another cross-sectional study, with 600 Chinese middle-aged and elderly women, individuals who slept ≤6 h/d had significantly lower total and regional BMD relative to those who slept 8 h/d [17]. In contrast, a cross-sectional study including 19,321 Japanese adults aged ≥50 years, reported that individuals with sleep duration of >8 h were more likely to have radial osteoporosis (diagnosis of osteoporosis was made if T-score of BMD was less than 70% of the young adult mean than those with short sleep duration (<6 h/d) (odds ratio [OR] = 1.35; 95% CI = 1.06–1.73) after adjustment for sex, age, BMI, current alcohol drinker, frequent exercise and some comorbidities [19]. A cross-sectional study conducted in China found that post-menopausal women, but not premenopausal women, with excessive total sleep had a higher likelihoods of osteoporosis (OR = 1.54 for >10 h/d vs. 8–9 h/d) [20]. Similar results were observed in another cross-sectional study including 8,688 Chinese adults, in which long or short sleep duration was associated with higher risk of osteoporosis in postmenopausal women, but not in men and premenopausal women [21]. In contrast, we found that men with ≥9h/d had lower BMD at the femoral neck than men with 8h/d sleep, but we did not observe significant differences in women. Further studies are warranted to examine whether these discrepancies reflect ethnicity difference or chance findings.

The Study of Osteoporotic Fractures and some other studies have suggested that disturbed sleep pattern (mostly referred to as short sleep or sleep inefficiency) could be a risk factor for fractures in older adults [32, 33]. However, these studies did not examine the potential effect of insomnia on BMD. To our knowledge, only one other study has addressed the association between insomnia and BMD. In the Tromsø Study, with 4,690 men and women, insomnia was not associated with distal BMD after adjusting for age, marital status, BMI, disability, physical activity, smoking and use of estrogen (the latter for women alone) [34]. These results are consistent with findings from the current study.

Strengths of our study include the measurement of BMD at multiple bone sites, a relatively larger sample size than most previous studies, and the inclusion of a number of important variables which could confound the potential association between sleep and BMD, such as BMI, smoking, alcohol use, physical activity, osteoporosis medication use, depression and plasma 25-hydroxyvitamin D concentration. We also measured several intermediate markers, including urinary cortisol and serum CRP and IL-6, which enabled us to explore the potential biological mechanisms underlying an association between sleep patterns and BMD. Further, this study is among the first to examine sleep disorders and bone health in a Hispanic population.

Our study also has several limitations. First, although larger than most previous studies, the sample size is still limited (we had 80% power to test the association of 0.27 between insomnia symptoms and BMD among women and 0.53 among men), precluding detection of small-to-modest effects on BMD. Second, assessment of insomnia symptoms and sleep duration was based on self-report, which could lead to misclassification. Thirdly, we did not measure sleep apnea, which may be associated with bone resorption [35]. However, we used self-reported snoring as a surrogate marker of sleep apnea. However, in a sensitivity analysis, excluding those with frequent snoring, we found similar non-significant associations between insomnia symptoms and BMD. Lastly, the cross-sectional design of this study precludes causality.

In conclusion, our study does not support the hypothesis that shorter sleep duration and insomnia symptoms are associated with lower BMD levels in older adults. However, our results should be interpreted with caution due to relatively small sample size and cross-sectional design of this study. Future studies with larger sample size, objective assessment of sleep pattern, and prospective design are needed before a firm conclusion regarding sleep patterns and BMD can be reached.
